# Whole blueberry protects pancreatic beta-cells in diet-induced obese mouse

**DOI:** 10.1186/s12986-019-0363-6

**Published:** 2019-05-22

**Authors:** Weixiang Liu, Yiping Mao, Jacob Schoenborn, Zhihong Wang, Guiliang Tang, Xiaoqing Tang

**Affiliations:** Department of Biological Sciences, Michigan Technological University, 1400 Townsend Dr, Houghton, MI 49931 USA

**Keywords:** Insulin sensitivity, Glucose tolerance, High fat diet, Islets, Insulin, Diabetes

## Abstract

**Background:**

Blueberry is rich in bioactive substances and possesses powerful antioxidant potential, which can protect against oxidant-induced and inflammatory cell damage and cytotoxicity. The aim of this study was to determine how blueberry affects glucose metabolism and pancreatic β-cell proliferation in high fat diet (HFD)-induced obese mice.

**Methods:**

Wild type male mice at age of 4 weeks received two different kinds of diets: high-fat diet (HFD) containing 60% fat or modified HFD supplemented with 4% (wt:wt) freeze-dried whole blueberry powder (HFD + B) for 14 weeks. A separate experiment was performed in mice fed with low-fat diet (LFD) containing 10% fat or modified LFD + B supplemented with 4% (wt:wt) freeze-dried whole blueberry powder. The metabolic parameters including blood glucose and insulin levels, glucose and insulin tolerances were measured.

**Results:**

Blueberry-supplemented diet significantly increased insulin sensitivity and glucose tolerance in HFD + B mice compared to HFD mice. However, no difference was observed in blood glucose and insulin sensitivity between LFD + B and LFD mice. In addition, blueberry increased β-cell survival and prevented HFD-induced β-cell expansion. The most important finding was the observation of presence of small scattered islets in blueberry treated obese mice, which may reflect a potential role of blueberry in regenerating pancreatic β-cells.

**Conclusions:**

Blueberry-supplemented diet can prevent obesity-induced insulin resistance by improving insulin sensitivity and protecting pancreatic β-cells. Blueberry supplementation has the potential to protect and improve health conditions for both type 1 and type 2 diabetes patients.

**Electronic supplementary material:**

The online version of this article (10.1186/s12986-019-0363-6) contains supplementary material, which is available to authorized users.

## Background

Type 2 diabetes develops as a consequence of a combination of insulin resistance and loss of pancreatic β-cell mass [1, 2]. Pancreatic β-cells are essential endocrine cellular units controlling blood glucose via the biosynthesis and secretion of insulin. In states of obesity and insulin resistance, insulin levels typically increase to maintain normal glucose tolerance. To compensate increased insulin demand, β-cell can adapt by increasing β-cell mass, leading to increased insulin production during obesity and insulin resistance. β-cell mass is defined as the total weight of β-cells within a pancreas and adaptive expansion of β-cell mass is accomplished primarily by increasing β-cell proliferation. However, during times of prolonged metabolic demand, inadequate β-cell function leads to increased apoptosis and a progressive loss of β-cell mass [3, 4]. Therefore, loss of functional β-cell mass is a hallmark of type 2 diabetes [5]. High concentrations of glucose, free fatty acids, reactive oxygen species (ROS) or proinflammatory cytokines converge toward a common cell death-signaling pathway and cause β-cell death during the development of type 2 diabetes [6–9]. Given β-cell failure during the progression to diabetes, more approaches are needed to preserve β-cell mass or enhance β-cell survival.

Blueberry has become a popular fruit that gained the interest of the public and scientific communities due to its role in maintaining and improving health. Blueberry is rich in bioactive compounds such as flavonoids and exhibit inhibitory effects on the induction of apoptosis [10–13]. In addition, blueberry possesses powerful antioxidant potential, which can consequently protect against oxidant-induced and inflammatory cell damage and cytotoxicity [14–17]. Many studies have reported that consumption of blueberries (in natural, dried, extracts or juice) were associated with a lower risk of type 2 diabetes and insulin resistance in rodents and humans [18, 19]. Particularly, blueberry has been found to inhibit lipogenesis, improve insulin sensitivity in muscle and adipose, and thus reduce the risk of developing type 2 diabetes [20–23]. Recent studies revealed that blueberry attenuated endothelial inflammation in diabetes, indicating its benefits in improving vascular complications associated with diabetes [24, 25]. However, whether blueberry affects β-cell function and growth were not evaluated in these studies. Only one report indicated that blueberry extracts significantly increased β-cell viability and inhibit triglyceride accumulation when incubating with INS832/13 β-cells [26].

In this article, blueberry-supplemented diet was applied to the mice fed with the combination of high-fat diet (HFD) to examine the effect of whole blueberry on β-cell mass and insulin production. Mice fed with HFD display obesity and hyperglycemia, which mimics the natural history of insulin resistance as well as metabolic features of human type 2 diabetes [27, 28]. We confirmed that supplement of blueberry in diet (HFD + B) significantly increased insulin sensitivity and glucose tolerance in mice. Furthermore, HFD + B diet enhanced β-cell survival and prevented HFD-induced β-cell expansion, which is a new discovery that will provide new insights into the effects of blueberry on β-cell function and expand our understanding the importance of blueberry in treating and preventing diabetes.

## Methods

### Animals and diets

The C57BL/6 J mice (000664, The Jackson Laboratory) were housed in a pathogen-free animal facility of Michigan Technological University with 12-h light / dark cycle and unlimited ad libitum water. Due to the fact that female mice are less susceptible to obesity and diabetes than female mice, male mice at 4 weeks of age were weighted and randomly assigned to one of the two groups (*n* = 5 mice/group) that received four different diets prepared at Research Diets Inc. (New Brunswick, NJ): 1) a high-fat diet (HFD, D12492, 60% kcal fat); 2) a modified HFD supplemented with 4% (wt:wt) freeze-dried whole blueberry powder (HFD + B); 3) a low-fat diet (LFD, D12450B, 10% kcal fat); 4) a modified LFD supplemented with 4% (wt:wt) freeze-dried whole blueberry powder (LFD + B) (see detailed compositions in Additional file 2: Table S1). The study utilized the whole blueberry powder, which was provided and shipped to Research Diets by the U.S. Highbush Blueberry Council following the specification by the funding agency. The energy from sucrose and total carbohydrates was adjusted to be equivalent between HFD and HFD + B, or between LFD and LFD + B. Diets were irradiated and stored at − 20 °C until use. All experiments were carried out in accordance with the approval by the Animal Care Committee at Michigan Technological University.

### Blood glucose and plasma insulin and glucagon levels

The body weight and blood glucose level were measured weekly starting at 5 week-old age. Mice were fasted for 16 h and fasting blood glucose was measured from 2 μl of tail vein blood with an Accuchek glucometer and glucose test strips (Abbott Diabetes Care). For plasma hormone assay, blood was harvested from orbital venous sinus using heparinized Natelson blood-collecting tubes. Plasma was prepared from blood collected by centrifugation at 4000 rpm for 5 min and kept in − 80 °C until use. Plasma insulin and glucagon levels were measured using ultrasensitive plasma insulin ELISA kit (10–1249-01, Mercodia) and glucagon ELISA kit (10–1271-01, Mercodia), respectively following the manufacturer’s instruction.

### Glucose tolerance test (GTT) and insulin tolerance test (ITT)

The glucose tolerance test was performed after a 16 h fast to 14-week-old mice. Mice were injected intraperitoneally with a glucose solution at a dose of 1.5 g/kg body weight. Blood samples were taken from the tail vein before and soon after glucose administration at 15, 30, 60, 90, and 120 min. Blood glucose levels were determined using glucometer and test strips. To assess the plasma insulin level during the GTT, the blood samples were obtained from the orbital vein at 0, 15, 30 min and Insulin levels were measured in plasma as described above. The area under the curve of GTT was calculated to analyze the glucose tolerance.

In the Insulin tolerance test (ITT), mice were fasted for 6 h and injected intraperitoneally with regular mouse insulin (I0516, Sigma) at 0.5 units/ kg body weight. Blood glucose was measured before (time = 0) and 15, 30, 45, 90 and 120 min after injection. The area under the curve of ITT was calculated to analyze the insulin sensitivity.

### β-Cell mass and islet size

Dissected mouse pancreas was fixed in 4% formaldehyde (pH 7.4) for 24 h at 4 °C, embedded in paraffin, and cut into 5-μm sections. The sections were then deparaffinised and stained for insulin (mouse anti-insulin, I2018, Sigma, 1:5000, overnight at 4 °C; secondary antibody: Alexa Fluor 488-conjugated anti-mouse from Invitrogen, A11001, 1:500, 1 h room temperature); nuclei were stained with DAPI in anti-fading mounting medium (H1200, Vector Labs). The whole pancreatic sections were captured by Leica whole slide scanner fluorescence microscopy. Islets were counted, and areas for every islet (Insulin^+^ area) and total pancreas were measured by the Image-Pro Premier software. β-cell mass (mg per pancreas) was calculated by obtaining the ratio of insulin^+^ area to total pancreas area of all scanned sections per animal and multiplied by the pancreas wet weight. To evaluate islet size distribution, islet size for each islet was obtained and the group of islet with different size was categorized. Five slides per animal (≥250 μm apart) were examined.

### β-Cell proliferation and in-situ TUNEL assay

Islet analysis after intraperitoneal injections of BrdU on seven consecutive days (100 μg/g Body Weight, 11669915001, Roche) was performed on 5 μm sections of paraffin-embedded pancreas approximately 50 μm apart. The slides were performed for normal immunohistochemistry staining using anti-BrdU (ab152095, 1:500, Abcam) and anti-insulin antibody. β-cells proliferation was expressed as the percentage of BrdU^+^/insulin^+^ cells per total number of insulin^+^ cells. At least 50 islets per slide and 3 different sections per animal were analyzed and normalized by total number of insulin positive cells.

Apoptosis was evaluated using transferase-mediated dUTP nick end labeling (TUNEL) with an in situ Cell Death Detection kit (11774425001, Roche) following supplier’s instructions. The sections were performed insulin staining followed after apoptosis staining as described above. The number of TUNEL-positive cells in islets was counted and the apoptotic index was expressed as the percentage of TUNEL^+^/insulin^+^ cells per total number of insulin^+^ cells.

### Quantitative real-time PCR for mRNA transcripts

Total RNA was extracted from islets using miRNeasy kit (217004, Qiagen) according to the manufacturer’s instructions and treated with DNase I (79254, Qiagen). The purity and quality of the extracted RNA were analyzed using NanoDrop and Bioanalyzer (Agilent 2100). A total of 250 ng high quality RNA (RIN ≥8) was reversely transcribed to cDNA with High Capacity cDNA Reverse Transcription Kit (4368814, Thermo Fisher). Quantitative RT-PCR (RT-qPCR) was performed with Power SYBR Green PCR Master Mix (4368706, Thermo Fisher) on a StepOnePlus™ Real-Time PCR System (Applied biosystem) using the following program: 10 mins at 95 °C, 40 cycles of 95 °C for 15 s and 60 °C for 1 min. All samples were run in duplicate, and the RNA expression of each gene was determined using relative comparison method (∆∆Ct), with Hypoxanthine guanine phosphoribosyl transferase (Hprt) mRNA as an internal standard.

### Statistical analysis

Data are expressed as means ± SD of three independent experiments. Statistical significance was determined by unpaired Student’s t-test (two-tailed) or oneway ANOVA with Tukey’s post hoc test with differences considered significant at *P* < 0.05 (marked as *) and *p* < 0.01 (marked as **).

## Results

### Blueberry-supplemented diet lowered plasma insulin level in HFD-fed mice

**Fig. 1:**
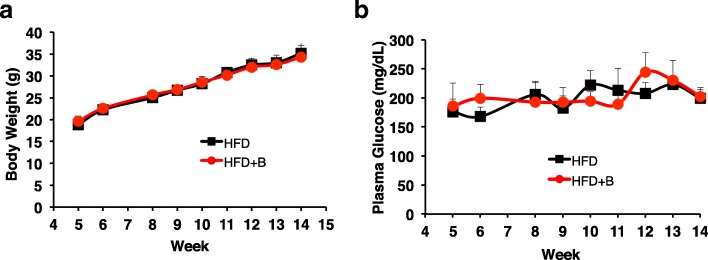
Effect of blueberry supplemented diet on body weight and blood glucose in HFD + B mice compared to HFD mice. **a** Weekly changes in body weight in mice fed with HFD or HFD + B between 4 and 14 weeks. **b** Blood glucose levels between HFD and HFD + B mice**.** *P < 0.05; **P < 0.01; *n* = 6–8 mice per group

Wild type mice fed with HFD exhibited a significant increase in body weight, blood glucose and insulin levels. To examine the effects of blueberry-supplemented diet on HFD-fed mice, the wild-type male mice, which are more susceptible to obesity and diabetes than female mice, received two different kinds of diets at 4 weeks of age: HFD or a modified HFD supplemented with 4% (wt:wt) freeze-dried whole blueberry powder (HFD + B). Compared to the mice fed with HFD, mice fed with HFD + B had no significant change in the body weight (Fig. 1a) and blood glucose level (Fig. 1b) throughtout 4 to15 weeks.

**Fig. 2:**
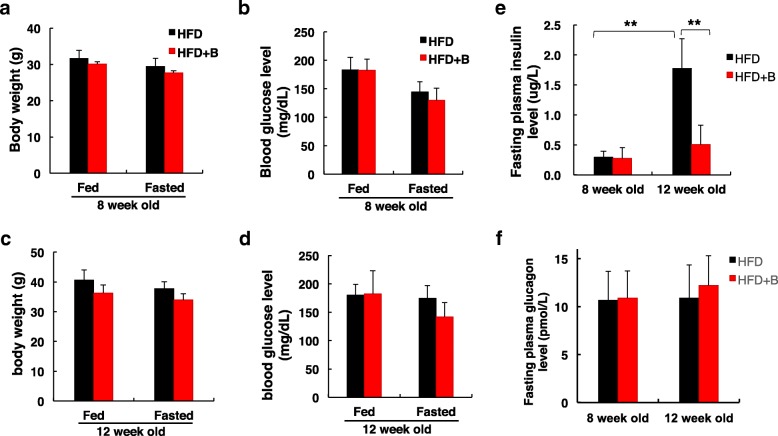
Effect of blueberry supplementation on plasma insulin and glucagon levels. **a** and **c** Body weight in HFD or HFD + B mice at 8 and 12 weeks of age. **b** and **d** Blood glucose levels between two groups. **e** Changes in fasting plasma insulin levels between two groups at 8 and 12 weeks of age. **f** Changes in fasting plasma glucagon levels between two groups after 16 h fast. Values are mean ± SD. ** *p* < 0.01

We further measured the blood glucose and plasma insulin levels before and after 16 h fasting of the two groups at week 8 and 12. No significant difference was observed between HFD + B and HFD mice regarding body weight (Fig. 2a, c) and blood glucose level (Fig. 2b, d). As expected, HFD feeding for 4 weeks significantly increased plasma insulin level and it was approximately 5-fold higher at week 12 compared to its level at week 8 (Fig. 2e). However, the addition of blueberry prevent the increase of plasma insulin (Fig. 2e). The plasma insulin level was much lower in HFD + B group than in the HFD group at 12 weeks of age, indicating that less insulin was required to maintain normal glucose level in HFD + B mice compared to HFD mice. In contrast, fasting plasma glucagon levels did not show significant differences between HFD + B and HFD groups (Fig. 2f).

### Blueberry supplementation improved insulin sensitivity and glucose tolerance in high-fat diet treated mice

**Fig. 3:**
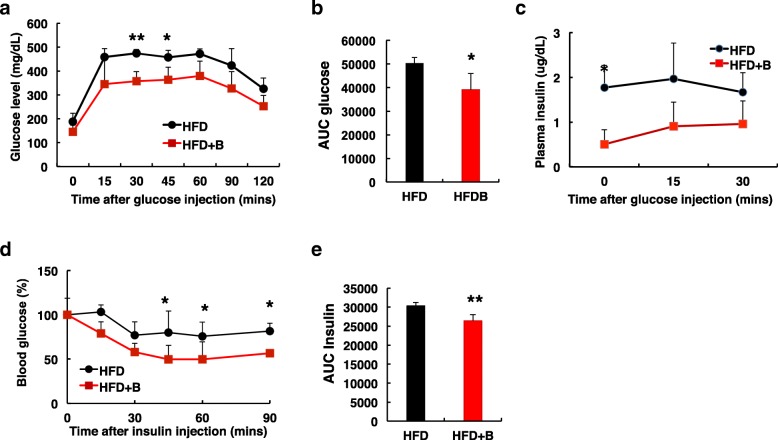
Diet supplemented with blueberry attenuated HFD-induced glucose intolerance and insulin resistance. **a** Glucose tolerance test (GTT) was performed in 12-wk-old mice fed with HFD or HFD + B after a 16 h fast. Blood glucose levels were determined at baseline and at the indicated times after glucose injection (1.5 g/kg body weight). **b** The area under the curve (AUC) during GTT. **c** The plasma insulin was measured at 0, 15 and 30 min after glucose injection (1.5 g/kg body weight). **d** Insulin tolerance test (ITT) was performed in 14-wk-old mice fed with HFD or HFD + B after a 6 h fast. Blood glucose levels were presented as the percentages of time-course blood glucose levels over the baseline level after intraperitoneal injection of insulin (0.5 U/kg body weight). **e** The area under the curve (AUC) during ITT. *P < 0.05; **P < 0.01; *n* = 4–8 mice per group

To examine the effect of the whole blueberry on metabolic features, we performed glucose tolerance test (GTT) in mice at 12 weeks of age. After glucose injection, as expected, blood glucose level reached above 400 mg/dL after 15 mins and remained high for 60 mins in HFD mice (Fig. 3a), confirming that 8 weeks of HFD feeding resulted in glucose intolerance. Compared to mice fed with HFD, mice fed with HFD + B cleared blood glucose much faster after glucose injection (Fig. 3a) and had significant lower AUC (area under the curve) level (Fig. 3b), suggesting that blueberry supplementation significantly enhanced glucose tolerance. Furthermore, compared to HFD mice, mice fed with HFD + B had significant lower plasma insulin level after overnight fast, as well as lower levels following glucose administration (Fig. 3c).

Insulin tolerance test (ITT) was further performed in the same groups of mice after 10 weeks of dietary treatment to assess the effects of blueberry on HFD-induced insuin resistance. As reported previously, mice fed with HFD displayed insulin resistance and had a blunted response to the exogenous insulin (Fig. 3d). However, mice fed with HFD + B responded to insulin with decreases in blood glucose after insulin injection. The area under the curve (AUC) during ITT was significant lower in mice fed with HFD + B compared to mice fed with HFD (Fig. 3e), suggesting that addition of blueberry increased insulin sensitivity.

We also examined the effect of blueberry-supplemented LFD (LFD + B) on blood glucose and insulin sensitivity. Compared to mice fed with LFD, no significant changes were observed on blood glucose and insulin sensitivity in mice fed with LFD + B for 10 weeks (Additional file 1: Figure S1). Taken together, these results suggested that blueberry can improve insulin sensitivity and glucose tolerance under HFD stress condition, and therefore delay insulin resistance caused by HFD feeding.

### Blueberry protected pancreatic β-cell function

**Fig. 4:**
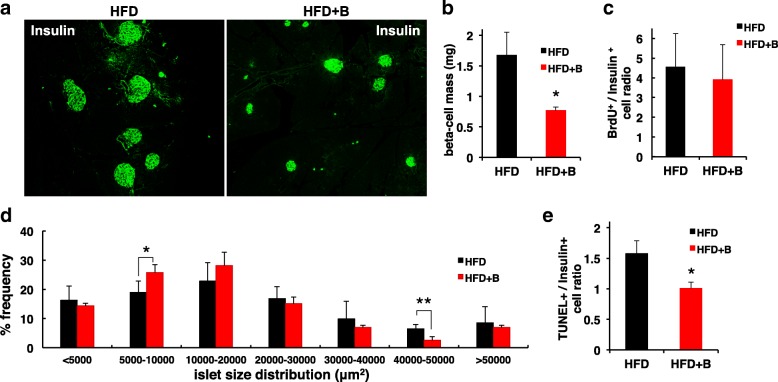
Blueberry supplementation preserved islet architecture. **a** A representative section of pancreas from 14-week-old mice fed with HFD or HFD + B was visualized by immunofluorescence after staining with anti-insulin (green) antibodies. **b** Changes in β-cell mass in 14-wk-old mice fed with HFD or HFD + B. **c** β-cell proliferation was evaluated by BrdU staining, the BrdU^+^/insulin^+^ cells to the total insulin^+^ cell ratio was quantified. **d** Size distribution of islets in HFD and HFD + B groups. Size distribution was categorized by percentage of total islets analyzed. Addition of blueberry to diet significantly increased the number of small islets in aggregates 5000–10,000 μm^2^ when compared to HFD. **e** Changes in β-cell apoptosis evaluated by TUNEL staining in 20-wk-old mice fed with HFD or HFD + B. The ratio of TUNEL^+^/insulin^+^ cells to the total insulin^+^ cells was quantified. Values are mean ± SD. *P < 0.05; **P < 0.01; *n* = 3–8 mice per group

To determine how blueberry attenuated HFD-induced insulin resistance, we performed morphometric analysis of the pancreas by immunofluorescence staining for insulin (Fig. 4a). Continuous HFD feeding induced a compensatory increase in β-cell mass due to increased insulin demand in mice fed with HFD. Compared to mice fed with HFD, mice fed with blueberry supplementation for 8 weeks exhibited a significant reduction in β-cell mass (Fig. 4b), suggesting that addition of blueberry prevented HFD-induced β-cell expansion. However, the β-cell mass reduction in HFD + B mice did not appear to be the result of reduced β-cell proliferation according to BrdU incorporation assay (Fig. 4c).

Accordingly, we analyzed islet area distribution in samples pooled from 3 animals per group (Fig. 4c). The number of small islets (defined as a diameter of an insulin-positive area 5000–10,000 μm^2^) was greater in HFD + B than in HFD mice. In contrast, the number of large islets (with diameter 40,000–50,000 μm^2^) was smaller in HFD + B mice compared to HFD mice. The data suggest that blueberry supplementation prevent HFD-induced β-cell mass expansion. In addition, blueberry supplementation may increase β-cell neogenesis or regeneration in HFD + B mice, as shown by the increased number of small islets.

To examine whether the reduction in β-cell mass in HFD + B mice was the result of increased apoptosis, the frequency of apoptotic cells was evaluated (Fig. 4e). However, few TUNEL signals were detected in HFD + B mice compared to HFD mice. β-cell apoptosis became apparent in mice at 20 weeks of age and we observed a modest increase of TUNEL staining in HFD mice when compared to HFD + B mice (Fig. 4e), indicating an important potential of increasing β-cell survival with blueberry supplementation.

Since plasma insulin levels were much lower in HFD + B mice compared to HFD mice, we reasoned whether the addition of blueberry affects insulin transcription. The expression of insulin and major β-cell specific transcription factors including Pdx-1 and MafA were examined in isolated islets and no significant changes were observed between the two groups (Data not shown). The data suggest that blueberry had no effect on insulin transcription, and the low fasting plasma insulin level in HFD + B mice mainly resulted from increased insulin sensitivity and reduced β-cell mass.

## Discussion

Our data demonstrated that blueberry-supplemented diet significantly increased insulin sensitivity in HFD-induced obesity mouse model, although the addition of blueberry did not prevent HFD-induced weight gain. Of note, similar findings have been observed in obese mice or diabetic mice that consumed a blueberry diet [29–31]. Similar results were also observed when obese or healthy adults consumed the whole blueberry or blueberry juices or capsules containing purified anthocyanins [21, 32, 33]. Although the underlying mechanisms remain unclear, much evidence suggests that blueberry inhibited the expression of nuclear factor κB, interleukin-6 (IL-6) and tumor necrosis factor alpha (TNFα) in the liver and abdominal adipose tissue, which may protect against adipocyte death and increase insulin sensitivity in obese-induced mice [30, 34, 35]. Contrarily, the anti-inflammatory effect was less pronounced in some animal studies and human [21, 36, 37]. New reports indicated that blueberry extracts can significantly inhibit inflammatory and apoptosis via activation of JAK1/STAT3 signaling [38], PPARγ activity [39] or survival PI3K/Akt and MAPK/ERK pathways [40].

Beside the improved insulin sensitivity, our results clearly indicated that blueberry supplementation significantly increased β-cell survival, improved glucose tolerance, and prevent β-cell mass expansion. These finding are important because the expansion of β-cell mass and the increase in insulin secretion are early signals of obesity and insulin resistance, which eventually lead to β-cell exhaustion, death and dysfunction [41, 42]. Thus, blueberry preserving β-cell structure and function will reduce the overwhelming burden of β-cells and prevent the development of obesity and diabetes. The possible mechanisms by which blueberry exerts its pancreatic protection involve enhancing β-cell survival by inhibition of cytokine expression and antioxidant stress. Previous in vitro study also showed that blueberry extracts significantly increased β-cell viability, reduced ROS level and improved the antioxidant defense system when culturing with INS832/13 β-cells [26].

The most important finding of the present study was the observation of more smaller scattered islets in blueberry treated obese mice, which may reflect neogenesis of new β-cells from pre-existing islet cells. β-Cell mass is regulated by three factors, proliferation, neogenesis (differentiation from precursor cells), and β-cell apoptosis [41]. It is believed that β-cells can regenerate through the replication of pre-existing β-cells or neogenesis from α- or duct cells inside the islets [43–46]. In human adults, the capacity for self-replication of remaining β-cells is too limited to result in a significant regeneration [47]. Therefore, enhancing neogenesis has a bigger potential to provide an increase of new β-cells that could then replicate further to provide enough β-cells to reverse diabetes [2, 48]. A number of herbs have been reported to induce the neogenesis of islets from the pre-existing islet cells [49–53]. The consumption of garlic induced a protective/ regenerative effect on β-cells [54]. Bitter melon also protected pancreatic damage and induced the renewal of β-cells in neonatal diabetic rats [55, 56]. Thus, the potentiality of blueberry in regenerating pancreatic β-cells will provide a new promising and welcome option for the patients who have lost functional islet cells.

## Conclusions

In summary, blueberry-supplemented diet significantly increased insulin sensitivity in HFD-induced obesity mouse model. In addition, diet supplemented with blueberry improved β-cell function by increasing β-cell survival and preventing β-cell mass expansion, which was a new discovery in this study. These findings provided new insights into the effects of blueberry on β-cell function and expand our understanding of the importance of blueberry in treating and preventing diabetes. Further studies are needed to define the molecular mechanisms underlying blueberry-mediated protective effects in islets and potential β-cell regeneration.

## Additional files

Additional file 1: **Figure S1.** Changes in blood glucose and insulin sensitivity in mice fed with LFD or LFD + B. **a **Weekly changes in blood glucose levels over 4–16 weeks. ** b** Insulin tolerance test (ITT) was performed in 16-week-old mice and blood glucose levels were assessed at the indicated times following an intraperitoneal injection of insulin (0.5 U/kg body weight). Values were represented as the percent of t = 0 glucose levels. *n* = 4–8 mice per group. (PDF 35 kb)

Additional file 2: **Table S1.** Composition of diets. (DOCX 109 kb)

## Abbreviations

Akt: Protein kinase B
AUC: Area under the curve
BrdU: 5-bromo-2′-deoxyuridine
DAPI: 4′, 6-diamidino-2-phenylindole
ERK: Extracellular-signal-regulated kinase
GTT: Glucose tolerance test
HFD + B: HFD supplemented with whole blueberry powder
HFD: High-fat diet
IL-6: Interleukin-6
ITT: Insulin tolerance test
JAK1: Janus Kinase 1
LFD + B: LFD supplemented with whole blueberry powder
LFD: Low-fat diet
MAPK: Mitogen-activated protein kinase
PI3K: Phosphoinositide 3-kinases
PPARγ: Peroxisome proliferator-activated receptor gamma
ROS: Reactive oxidative species
STAT3: Signal transducer and activator of transcription 3
TNFα: Tumor necrosis factor alpha
TUNEL: Terminal deoxynucleotidyl transferase-mediated dUTP nick-end labeling
