# Supplementary figures and images for: Whole blueberry protects pancreatic beta-cells in diet-induced obese mouse
Source: Nutr Metab (Lond). 2019 May 22;16:34. doi: 10.1186/s12986-019-0363-6 (PMC6530052; doi:10.1186/s12986-019-0363-6)

**A**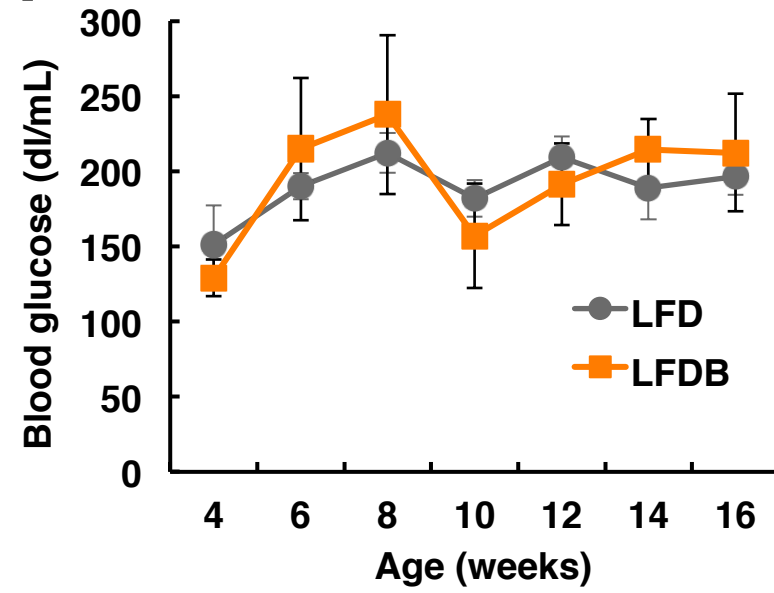**B**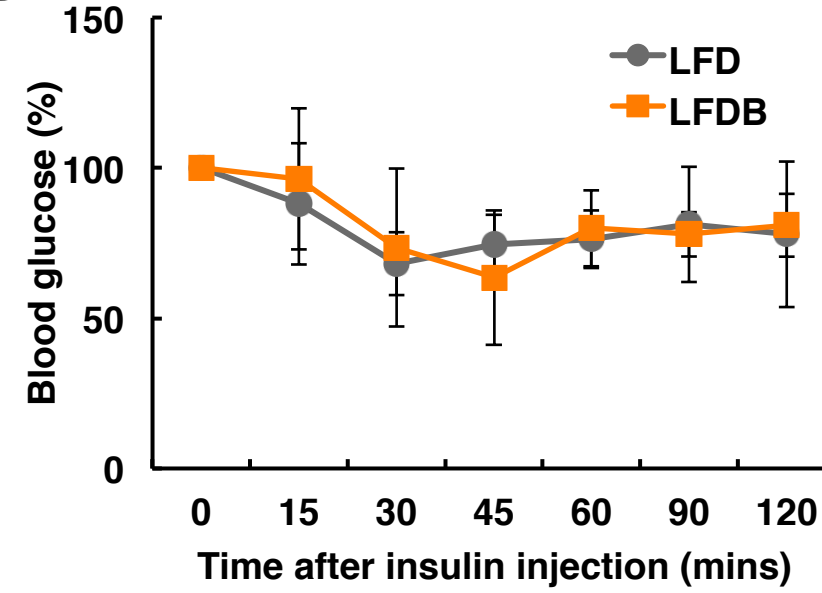

Supplement: Supplementary file 1 — Figure S1. Changes in blood glucose and insulin sensitivity in mice fed with LFD or LFD + B. a Weekly changes in blood glucose levels over 4–16 weeks. b Insulin tolerance test (ITT) was performed in 16-week-old mice and blood glucose levels were assessed at the indicated times following an intraperitoneal injection of insulin (0.5 U/kg body weight). Values were represented as the percent of t = 0 glucose levels. n = 4–8 mice per group. (PDF 35 kb) [file 12986_2019_363_MOESM1_ESM.pdf]
