# Supplementary material for: Whole blueberry protects pancreatic beta-cells in diet-induced obese mouse
Source: Nutr Metab (Lond). 2019 May 22;16:34. doi: 10.1186/s12986-019-0363-6 (PMC6530052; doi:10.1186/s12986-019-0363-6)
Supplement: Supplementary file 2 — Table S1. Composition of diets. (DOCX 109 kb) [file 12986_2019_363_MOESM2_ESM.docx]

**Table S1** Composition of diets

| **Product #** | **HFD** | **HFD+B** | **LFD** | **LFD+B** |
| --- | --- | --- | --- | --- |
|  | **gm% kcal%** | **gm% kcal%** | **gm% kcal%** | **gm% kcal%** |
| Protein  Carbohydrate  Fat  kcal/gm  **Ingredient**  Casein, 80 Mesh  L-Cystine  Corn Starch  Maltodextrin 10  Sucrose  Cellulose, BW200  Soybean Oil  Lard  Mineral Mix, S10026  DiCalcium Phosphate  Calcium Carbonate  Potassium Citrate, 1H2O  Vitamin Mix, V10001  Choline Bitartrate  Freeze Dried Blueberry Powder  FD&C Yellow Dye #5  FD&C Red Dye #40  FD&C Blue Dye #1  Total | 26.2 20  26.3 20  34.9 **60**  5.24  **gm kcal**  200 800  3 12  0 0  125 500  68.8 275.2  50 0  25 225  245 2205  10 0  13 0  5.5 0  16.5 0  10 40  2 0  0 0  0 0  0 0  0.05 0  773.85 4057 | 25.2 20  25.3 20  33.5 **60**  5.03  **gm kcal**  200 800  3 12  0 0  125 500  68.8 275.2  50 0  25 225  245 2205  10 0  13 0  5.5 0  16.5 0  10 40  2 0  32.3 0  0 0  0.05 0  0 0  806.15 4057.2 | 19.2 20  67.3 70  4.3 **10**  3.85  **gm kcal**  200 800  3 12  506.2 2024.8  125 500  68.8 275.2  50 0  25 225  20 180  10 0  13 0  5.5 0  16.5 0  10 40  2 0  0 0  0.04 0  0 0  0.01 0  1055.1 4057 | 18.7 20  65.3 70  4.1 **10**  3.73  **gm kcal**  200 800  3 12  506.2 2024.8  125 500  68.8 275.2  50 0  25 225  20 180  10 0  13 0  5.5 0  16.5 0  10 40  2 0  32.3 0  0 0  0.025 0  0.025 0  1087.35 4057 |
| Blueberry Powder (%) | 0 | 4.0 | 0 | 3.0 |

The composition for all diets including high-fat-diet (HFD), low-fat-diet (LFD) and blueberry-supplemented HFD+B and LFD+B were provided by Research Diets, Inc.
